# Chitosan–Zinc(II) Complexes as a Bio-Sorbent for the Adsorptive Abatement of Phosphate: Mechanism of Complexation and Assessment of Adsorption Performance

**DOI:** 10.3390/polym10010025

**Published:** 2017-12-25

**Authors:** Maryam Roza Yazdani, Elina Virolainen, Kevin Conley, Riku Vahala

**Affiliations:** 1 Water and Wastewater Engineering Research Group, School of Engineering, Aalto University, P.O. Box 15200, FI-00076 Aalto, Finland; elina.virolainen@gmail.com (E.V.); riku.vahala@aalto.fi (R.V.); 2 COMP Centre of Excellence, Department of Applied Physics, School of Science, Aalto University, FI-00076 Aalto, Finland; kevin.conley@aalto.fi

**Keywords:** zinc–chitosan complexes, characterization, bio-sorbent, phosphate, adsorption, mechanism, thermodynamic

## Abstract

This study examines zinc(II)–chitosan complexes as a bio-sorbent for phosphate removal from aqueous solutions. The bio-sorbent is prepared and is characterized via Fourier Transform Infrared Spectroscopy (FT-IR), Scanning Electron Microscopy (SEM), and Point of Zero Charge (pH_PZC_)–drift method. The adsorption capacity of zinc(II)–chitosan bio-sorbent is compared with those of chitosan and ZnO–chitosan and nano-ZnO–chitosan composites. The effect of operational parameters including pH, temperature, and competing ions are explored via adsorption batch mode. A rapid phosphate uptake is observed within the first three hours of contact time. Phosphate removal by zinc(II)–chitosan is favored when the surface charge of bio-sorbent is positive/or neutral e.g., within the pH range inferior or around its pH_PZC_, 7. Phosphate abatement is enhanced with decreasing temperature. The study of background ions indicates a minor effect of chloride, whereas nitrate and sulfate show competing effect with phosphate for the adsorptive sites. The adsorption kinetics is best described with the pseudo-second-order model. Sips (*R*^2^ > 0.96) and Freundlich (*R*^2^ ≥ 0.95) models suit the adsorption isotherm. The phosphate reaction with zinc(II)–chitosan is exothermic, favorable and spontaneous. The complexation of zinc(II) and chitosan along with the corresponding mechanisms of phosphate removal are presented. This study indicates the introduction of zinc(II) ions into chitosan improves its performance towards phosphate uptake from 1.45 to 6.55 mg/g and provides fundamental information for developing bio-based materials for water remediation.

## 1. Introduction

Phosphate is an important element for many natural organisms, yet in high concentrations, it can cause serious eutrophication in natural waters [1,2,3,4,5]. In eutrophic waters, the exceeding amount of nutrients leads to the excessive growth of plants and algae. This phenomenon reduces the dissolved oxygen in the water, which disturbs the natural balance of organisms, and causes, e.g., mass fish death [1,6]. Eutrophication raises the costs of water treatment, weakens the recreational use of waters and enables the growth of algal blooms that produce harmful cyanotoxins [7]. Phosphorus dissolves into natural waters from weathered rocks, peat land and forests, and it gets into the communal wastewaters through agriculture, human settlement and industry [7,8]. Miettinen et al. [8] have studied the connection of phosphorus and bacterial growth in drinking water sources and showed that in both surface and ground waters the addition of phosphorus strongly contributes to the growth of heterotrophic bacteria. 

While the maximum phosphate concentration set by US Environmental Protection Agency (EPA) is 0.05 mg/L [9], even a concentration of 0.02 mg/L can cause major eutrophication [6]. Preserving the aquatic life from phosphate contamination requires new phosphate removal techniques [2,10]. The commonly practiced methods for phosphate abatement include chemical precipitation and biological removal, but they are problematic in different ways. These treatment processes usually fail to meet the standard levels set for phosphate or even to decrease it to below 10 mg/L [11]. In addition, chemical precipitation is a relatively expensive method, requiring the storage and transportation of chemical reagents and producing considerable sludge waste. Biological phosphorus removal on the other hand is relatively sensitive to water conditions, which decreases it reliability. Compared to these techniques, adsorption technology offers a simple and low cost option [7]. Recently, engineered adsorbents have attracted a great deal of attention as alternatives for common yet expensive adsorbents, e.g., activated carbon. This new type of adsorbents usually comprises two or more constituents, one of which acts as a support matrix [12]. For instance, impregnated polymers with metal oxides [3] and biomass modified with mesoporous materials [6] have previously been studied as phosphate adsorbents. 

Chitosan (CTS) is a biopolymer emerging in the adsorption process and is derived from chitin, the second most plentiful natural polymer after cellulose [13,14,15]. It has many advantages as a bio-sorbent, such as good adsorption capacity, biodegradability, and biocompatibility [15,16,17,18,19]. As it is extracted from crustacean waste, it is eco-friendly and cost-effective compared to commonly employed adsorbents. Despite its good adsorption capacity for a wide range of pollutants, CTS provides a low affinity towards oxyanions mainly because of the p*K*_a_ value of its electron-donor functional sites, viz. –NH_2_ [12]. To overcome this obstacle, the introduction of metal ions to CTS has recently been practiced [15]. Metal ions, e.g., zinc(II) [20] and copper(II) [12], are capable of forming complexes with the functional groups on the CTS chain, and consequently the complexed metal ions on the CTS structure can link with other ligands including oxyanions. Among different metal ions, zinc (Zn) can easily chelate with CTS, which has made it the focus of many studies on its application for metal ions separation [20], waste management [21], antibacterial, and medical aspects [20]. However, to date, there is limited information on Zn(II)–CTS complexes as adsorptive media for the removal of oxyanions like phosphate. Along with the research conducted on the antimicrobial activity, thermal degradation and pyrolysis characteristics of CTS complexation with zinc(II) [20,21], a study on its application as a bio-sorbent provides deeper knowledge of the complexation and enable a better performance in the application stage towards specific goals, e.g., phosphate removal. Thus, a prospect of using Zn(II)–CTS bio-sorbent for phosphate removal was conceptualized in this study.

Here, we develop Zn(II)–CTS bio-sorbents for the removal of phosphate and study their response to adsorbent dose, phosphate concentration, solution pH, water temperature, contact time and competing ions (Cl^−^, NO_3_^−^, SO_4_^2−^) effects. The bio-sorbent dosage is optimized and compared with those of plain CTS and the composites produced from both zinc oxide (ZnO) and nano-sized zinc oxide (nano-ZnO) embedded in CTS to indicate its better performance towards phosphate abatement. The composition, morphology and interface of the Zn(II)–CTS are characterized by Fourier transform infrared spectroscopy (FT-IR), scanning electron microscopy (SEM) and determination of the pH of Point of Zero Charge (pH_PZC_). 

## 2. Experimental 

### 2.1. Materials

Chitosan was supplied by Acros Organics of Thermo Fisher Scientific Inc., Geel, Belgium. The degree of deacetylation (DDA) of chitosan was 84 ± 1% determined via acid–base titration [22,23] and its average molecular weight ($\bar{M_{W}}$) was 153.3 kD measured via determination of intrinsic viscosity as described previously [24,25]. Zinc oxide (>99%) and zinc chloride (>98%) were purchased from Merck chemicals (Darmstadt, Germany) and zinc oxide nano-powder (<50 nm particle size (BET), >97%) was supplied by Sigma Aldrich, Darmstadt, Germany. A stock solution with the concentration of 1000 mg/L was made via weighing an accurate amount of potassium dihydrogen phosphate (KH_2_PO_4_) and dissolving it in reverse osmosis water. Different dilutions in the range of 1–15 mg/L were prepared daily before each adsorption set.

### 2.2. Analysis Methods and Instruments

The pH of phosphate solutions was measured according to the SFS-EN ISO 10523 (dated 2012) with WTW inoLab pH 720-meter and probe Sentix 81 Plus. The pH meter was calibrated before each use. Phosphate concentration was measured according to the SFS-EN ISO 15681-1 (dated 2005) via flow analysis (FIA) and spectrometric detection using tin chloride method on a FOSAS Tecator, FIAstar 5000 Analyzer and Sampler 5027. 

### 2.3. Adsorbent Preparation and Characterization

This study investigated three different bio-sorbents developed from CTS and zinc compounds; Zn(II)–CTS, ZnO–CTS and nano-ZnO–CTS. The Zn(II)–CTS bio-sorbent was prepared as follows: dissolved CTS (1 g) into 0.1 M acetic acid (100 mL) was agitated for 24 h at room temperature at a speed of 180 rpm to achieve a thoroughly homogenous solution. Then 0.34 g of zinc chloride dissolved into 50 mL of reverse osmosis water was slowly added into the CTS solution while continuously stirring until a homogenous solution was achieved. This solution was heated to 80 °C for 1 h with continuous stirring. After cooling to room temperature, the solution was pumped into 0.5 M NaOH solution (50 mL per 250 mL NaOH) using a syringe pump to develop the bio-sorbent in the form of beads. The product was immersed in the NaOH solution overnight. The final solid product was separated, rinsed with reverse osmosis water to reach neutral pH, and dried in an oven at 25 °C. ZnO–CTS was prepared in a similar way. The suspension of ZnO (0.2 g) in 0.1 M acetic acid (100 mL) was agitated for 24 h at room temperature and at the speed of 180 rpm before mixing with the CTS. The ZnO suspension was mixed with CTS solution and agitated for another 24 h at room temperature. The final ZnO–CTS was pumped into NaOH solution. Nano-ZnO–CTS was prepared as ZnO–CTS, but the pH of nano-ZnO/acetic acid suspension was set to 4. The mass of zinc–compounds, ZnCl_2_ and ZnO, was determined in a way that the bio-sorbent products contained an equal amount of Zn.

The FT–IR analysis of the developed bio-sorbent was performed on a Thermo scientific Nicolet iS50 FT-IR spectrometer with a PIKE Gladi-ATR. SEM was performed with ZEISS Sigma VP (Jena, Germany) using 2 kV acceleration voltage, detecting secondary electrons. Samples were attached to an aluminum stub using carbon tape, and sputter coated with platinum using Emitech K100X for 90 s and 30 mA coating current to prevent charging effects. The pH drift method was employed to determine the pH_PZC_ of the Zn(II)–CTS surface using 20 mL of 0.1 M NaCl in a series of solutions for which pH was adjusted within the range of 3 to 12. After the initial pH of NaCl solutions was adjusted using NaOH and HCl, 0.01 g of the bio-sorbent was added to each of them and their final pH was measured after 24 h. The pH_PZC_ was noted at the pH where the final pH equals the initial pH [26].

### 2.4. Adsorption Experiments

All batch experiments were conducted on a shaker at the speed of 180 rpm using 50 mL phosphate solutions and known amount of bio-sorbent. The pH of phosphate solution was set to 4 unless otherwise mentioned. After the required contact time, the solutions were filtrated with Sartorius Minisart 45 μm filters and analyzed for final phosphate concentration. All experiments excluding the isotherm tests were performed at room temperature for 24 h. The optimum adsorbent dose was determined by conducting the experiment with five different doses in range of 0.1–2 g/L. The pH effect was studied with adjusting the pH at different values between 4 and 12 using HCl and NaOH. The effect of contact time was studied at different time intervals between 1 min to 48 h. Phosphate solutions with initial concentration from 1 to 15 mg/L were used to study adsorption isotherm at 20, 25, and 30 °C. NO_3_^−^, SO_4_^2−^ or Cl^−^ was added to investigate the effect of competing ions on phosphate adsorption. All the adsorption experiments were carried out in two or three replicates when high standard deviation values were noticed for the removal percentage. The average of the replicates and the standard deviation values are reported accordingly. Phosphate removal and adsorption capacity were calculated with the following equations:

(1) $$\mathrm{Removal}\%=\frac{\left(C_{0}-C_{t}\right)}{C_{0}}\times 100$$

(2) $$q_{t}\;\left(\mathrm{mg}/g\right)=\frac{C_{0}-C_{t}}{m}\times V$$

where *C*_0_ (mg/L) is the initial phosphate concentration, *C_t_* (mg/L) the phosphate concentration at time *t*, *V* (L) the solution volume, and *m* (g) the adsorbent mass.

## 3. Results and Discussion

### 3.1. Characterization of Zn(II)–CTS Bio-Sorbent

#### 3.1.1. FT-IR Analysis

**Figure 1:**
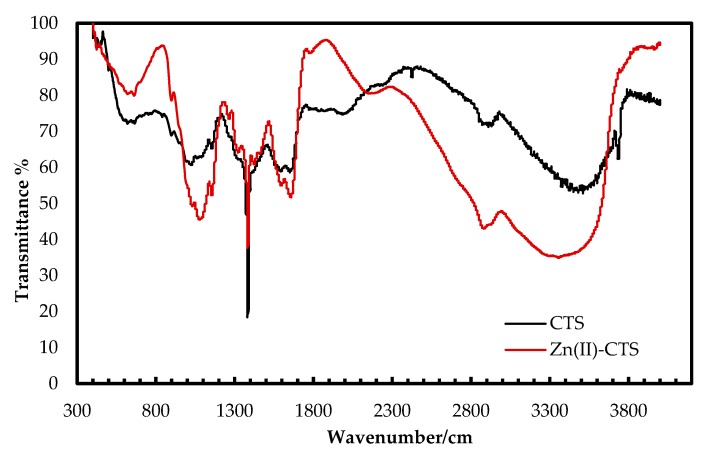
Fourier transform infrared (FT-IR) spectra of CTS and Zn(II)–CTS.

The FT–IR spectra of Zn(II)–CTS complexes and plain CTS were analyzed to determine the compositional differences as seen in Figure 1. The broad characteristic peak in the region 3100–3600 cm^−1^, related to the stretching vibration of –NH_2_ and –OH groups of CTS, are shifted to lower wavenumbers on the spectrum of Zn(II)–CTS. The O–H stretching band at 3740 cm^−1^ on the spectrum of CTS has moved to 3760 cm^−1^ with a lower intensity on the spectrum of Zn(II)–CTS. The peak at 1640 cm^−1^, related to the –NH_2_ bending vibration of CTS, is shifted to higher frequencies (1660 cm^−1^) for Zn(II)–CTS indicating the –NH_2_ and –OH groups on the CTS backbone have complexed with Zn(II). The band at 1090 cm^−1^ representing the secondary –OH of CTS is moved to 1080 cm^−1^ along with a higher intensity on the spectrum of Zn(II)–CTS. The band shift from 1090 cm^−1^ to 1080 cm^−1^ is characteristic of the coordination of –OH with Zn [20]. The peaks at 2920, 2880, 1600, 1380, 1080 and 620 cm^−1^ are assigned to methylene C–H_2_ and methyl C–H stretching vibrations, N–H groups, C–H asymmetric bending, C–O alcohol stretching and O–H bending (out-of-plane), respectively. The vibrational bands at 1780 and 898 cm^−1^ correspond to the C=O band and glucopyranose ring of CTS, respectively. The peak at 620 cm^−1^, assigned to the hydroxyl groups of CTS, has moved to 650 cm^−1^ on the spectrum of Zn(II)–CTS. Moreover, the appearance of peaks around 450 and 570 cm^−1^ on the spectrum of Zn(II)–CTS are characteristics of the stretching vibrations of Zn–O and Zn–N [20,27]. 

#### 3.1.2. SEM Analysis

**Figure 2:**
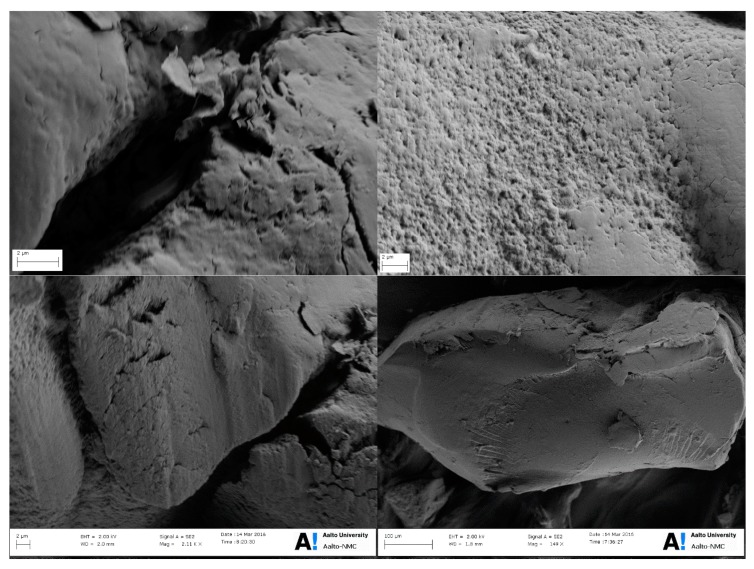
Scanning electron microscopy (SEM) images of Zn(II)–CTS beads at different magnifications (2 µm and 100 µm).

The surface morphology of Zn(II)–CTS was studied via scanning electron microscopy. Figure 2 indicates that the developed Zn(II)–CTS bio-sorbent shows an irregular and rough surface including micro-pores and small fractures, into which the oxyanions can penetrate and better access the internal functional adsorptive sites. Porosity enables the second phase adsorption according to the intra-particle diffusion theory, which is further discussed in kinetics modelling. The bio-sorbent particles have an irregular bead-like shape (granular) as seen in Figure 2.

#### 3.1.3. Determination of pH_PZC_

**Figure 3:**
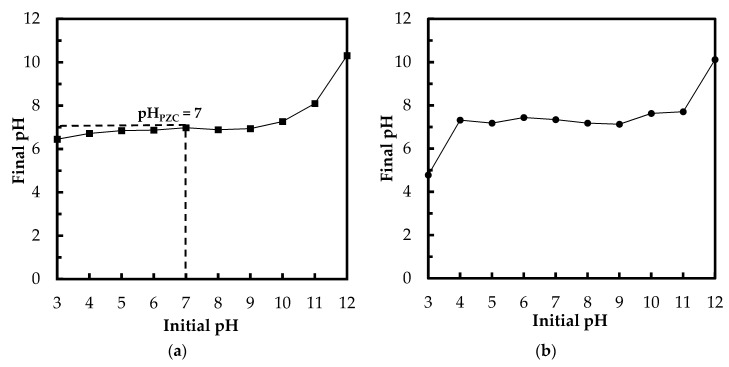
(**a**) The determination of point of zero charge (pH_PZC_) using 0.1 M NaCl solutions (drift method); and (**b**) change of pH in the 5 mg/L PO_4_^3−^/Zn(II)–CTS solutions (Experimental conditions: 0.01 g of Zn(II)–CTS; 20 mL solution volume; contact time 24 h).

Point of zero charge plays a key role in the surface science of environmental interfaces, where it indicates how easily adsorptive materials are able to adsorb the ions of target pollutants. The pH_PZC_ is the pH when the charge on the adsorbent surface is zero. Figure 3a depicts the results of the pH_PZC_ determination via drift method (NaCl solutions). The pH_PZC_ of Zn(II)–CTS bio-sorbent was determined to be approximately 7, which is a reasonable pH_PZC_, given that the p*K*_a_ of CTS ranges from 6.3 to 7.2. At solution pH above the pH_PZC_, the surface of the bio-sorbent is negatively charged, whereas at pH values below pH_PZC_, the surface becomes positively charged and oxyanions adsorption happens due to electrostatic interaction of anions with the positively charged surface of the bio-sorbent. Hence, there is an increase in phosphate adsorption when the solution pH is lower than pH_PZC_ [3,26]. A similar experimental set was conducted with PO_4_^3−^ solutions (Figure 3b). The final pH converged to around 7 when the initial pH was set from 4 to 11. For the solution with the initial pH of 3, the final pH remained below the pH_PZC_ which can be due to the abundant H^+^ ions preventing the pH raise induced by the bio-sorbent. For the sample with initial pH of 12, the final pH was 10.1, higher than pH_PZC_, which happens due to the excessive amount of OH^−^ ions. Above pH_PZC_, the adsorption of anions is hindered by the negatively charged surface of Zn(II)–CTS.

### 3.2. Phosphate Adsorption Studies

#### 3.2.1. Effect of Bio-Sorbent Dose

**Figure 4:**
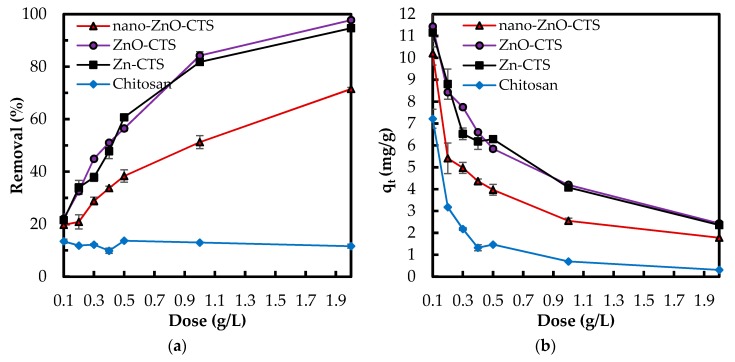
(**a**) Effect of adsorbent dose on (**a**) phosphate removal percentage; and (**b**) phosphate adsorption capacity (mg/g) (Experimental conditions: 5 mg/L phosphate concentration; natural pH; 50 mL solution).

Adsorbent dose was optimized for three zinc compound–CTS bio-sorbents, Zn(II)–CTS, ZnO–CTS and nano-ZnO–CTS, and additionally for a plain CTS sample. Different adsorbent doses varying between 0.1 to 2 g/L were studied. Similar trends in the results of phosphate removal percentage and adsorption capacity (mg/g) were observed for all three composites. As the amount of absorbent was increased, the removal percentage increases (Figure 4). This is because the growing adsorption surface area increases the active adsorptive sites on the surface. With the adsorbent dose of 0.1 g/L, all three bio-sorbents reached a removal percentage in the range of 19.7–21.7%. After this, the removal efficiency of nano-ZnO–CTS grew less than those of ZnO–CTS and Zn(II)–CTS. At the maximum adsorbent dose, 2 g/L, the removal percentage of nano-ZnO–CTS was 71.5% whereas for ZnO–CTS and Zn(II)–CTS 97.75% and 94.65%, respectively. When comparing the nano-ZnO–CTS to the other two bio-sorbets, the lower adsorption performance, e.g., removal percentages and corresponding capacities in Figure 4, along with higher standard deviations may be caused by the aggregated nano ZnO particles in the nano-ZnO–CTS beads. In comparison, the plain CTS sample (Figure 4a) proved to be relatively unsuccessful with only 10% average removal, which is mainly due to the p*K*_a_ value of its electron-donor functional groups, viz. –NH_2_. The formation of new binding sites by Zn(II) ions within the CTS matrix is followed by the coordination of the phosphate anions to zinc(II). These chelating sites are unavailable to phosphate anions in the plain CTS, proved by the low performance of CTS in Figure 4. This observation confirms the improving effect of zinc(II) complexes in the adsorption performance of CTS towards phosphate oxyanions.

As seen in Figure 4b, the adsorption capacity *q_t_* decreases for all four adsorbents with increasing dose. The capacity decreased for ZnO–CTS from 12.37 to 2.43 mg/g, for Zn(II)–CTS from 10.99 to 2.37 mg/g, and for nano-ZnO–CTS from 10.21 to 1.78 mg/g. This reduction can happen due to (I) a gap in the flux of phosphate concentration gradient between the concentrations in the liquid phase and on the solid surface, causing the amount of phosphate adsorbed onto the unit weight of adsorbent to decrease with the increasing dose [28], and/or (II) the increase in the adsorbent dose for a given amount of phosphate in the solution results in the unsaturation of adsorbent sites. In addition, more adsorptive sites become occupied, hence creating an increasing repulsion between the molecules in the solution and adsorbed on the surface [4].

The bio-sorbent/liquid ratio was set to 0.5 g/L for the rest of adsorption studies to account for the balance between the lower removal percentage at lower dosage and lower adsorption capacity (*q*) at higher dosage.

#### 3.2.2. Effect of pH

**Figure 5:**
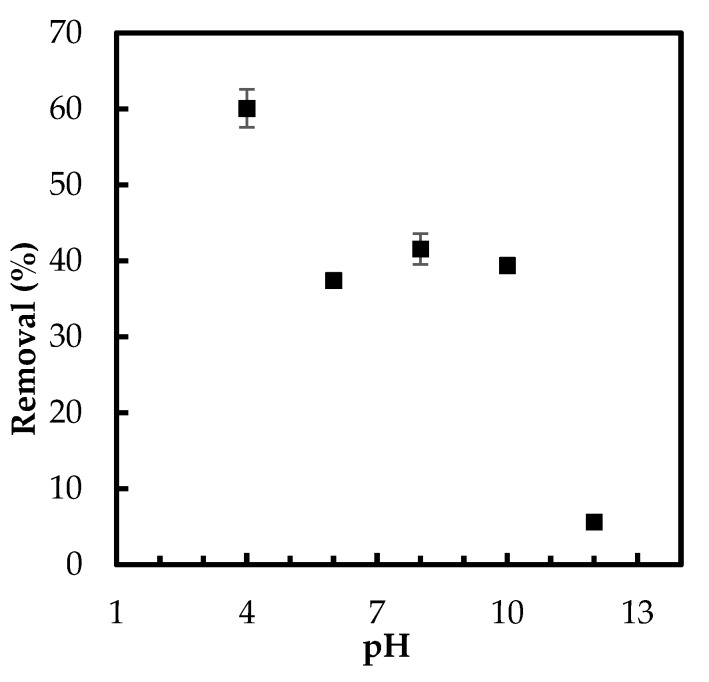
Effect of pH on phosphate removal by Zn(II)–CTS complex (Experimental conditions: 0.5 g/L adsorbent dose; 5 mg/L initial phosphate concentration).

The role of pH in phosphate uptake by the developed Zn(II)–CTS bio-sorbent was studied in five different pH values. The binding of phosphate oxyanions to the bio-sorbent occurred more efficiently in acidic medium, as seen in Figure 5. This enhancement of the removal percentage is consistent with previous studies of adsorptive abatement of phosphate [3]. 

The decrease in the phosphate adsorption onto Zn(II)–CTS by shifting pH from acidic to basic conditions can be attributed to the change in the distribution of phosphate species (H_2_PO_4_^−^, HPO_4_^2−^, and PO_4_^3−^) and the decrease in surface protonation of the bio-sorbent. Additionally, in the basic solution, the hydroxyl ions may compete with phosphate adsorption [4]. High pH alter the aqueous form of phosphate species from H_2_PO_4_^−^ to HPO_4_^2−^. The latter species is more resistant to the uptake by the surface hydroxyl groups. The limited adsorption at higher pH can also be caused by the excess amount of OH^−^ ions and deprotonation of Zn(II)–CTS surface, leading to a decrease in the interaction between the surface of Zn(II)–CTS and phosphate anions [28]. On the other hand, lower acidic condition protonates the surface functional groups of Zn(II)–CTS complexes, as discussed in the section related to pH_PZC_, resulting in higher phosphate uptake due to the electrostatic attraction. As showed in Figure 3b, there is a shift in the final pH of the phosphate solutions from the initial pH. The solution with an initial pH of 12, for instance, had a final pH of 10, which is higher than pH_PZC_ of the bio-sorbent. The surface is negatively charged above pH_PZC_ and consequently repels phosphate oxyanions. For the initial pH ranging 6–10, the final pH of the solutions shifted to around pH_PZC_, 7, resulting in a neutral surface of the bio-sorbent. Therefore, the anions of phosphate were able to easily move to the surface and finally chelate with the Zn(II) sites of the bio-sorbent. Because the surface is neutral between pH 6 and 8 (Figure 5), the slightly higher removal at pH 8 may be due to more readily accessible Zn(II) adsorptive sites. Furthermore, considering the kinetics of transport into a charged pore, salt will screen the charges in the pores allowing for faster transport into the pores. Therefore, there is a faster/better adsorption. The solution with initial pH 4 showed the highest phosphate removal implying a positive surface charge of the bio-sorbent, which favored the oxyanion removal. 

#### 3.2.3. Adsorption Kinetics

**Figure 6:**
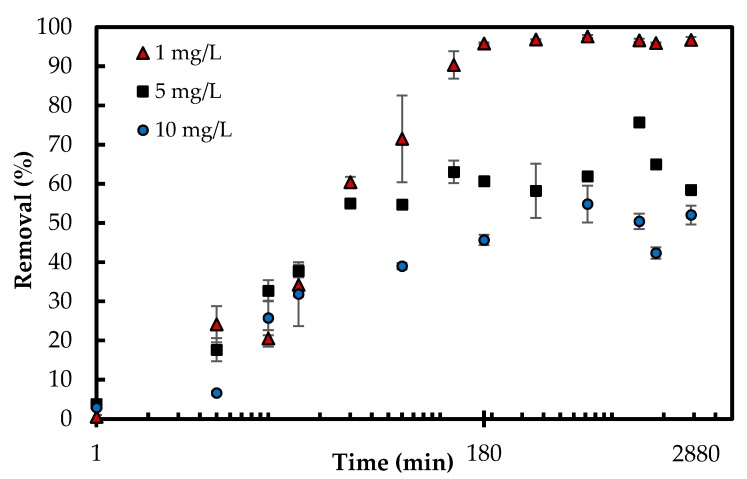
Effect of contact time on phosphate removal percentage (Experimental conditions: 0.5 g/L Zn(II)–CTS dose; 1, 5 and 10 mg/L initial phosphate concentration; pH 4).

When it comes to the economical design of an adsorption process, the time for the system to reach equilibrium is an essential parameter [4]. The contact time was studied within 13 different time intervals from 1 min up to 48 h for three different phosphate concentrations (1, 5, 10 mg/L). The adsorption equilibrium and maximum capacities of phosphate adsorption were obtained within 180 min. All three sets of experiments increased the removal percentage during the first 180 min, which was followed by a plateau. The concentration gradient of phosphate in the solution and the existence of the higher number of available active sites on the surface drive the increase in adsorption. The maximum phosphate removal percentages for 10, 5 and 1 mg/L were 54.84%, 75.70% and 97.63%, respectively (Figure 6). The corresponding adsorption capacity (mg/g) for those percentages were 10.99, 7.39 and 2.00 mg/g, respectively. The decrease in removal by increasing concentration from 1 to 10 mg/L is expected as there are more molecules of adsorbate in the solution when the concentration increases so that the denominator of the percentage is greatly increased, resulting in a lower percentage. Furthermore, the adsorbent surface has a saturation stage (Langmuir monolayer coverage 5.33 mg/g discussed in the adsorption isotherm section). Once the surface is covered, the adsorption kinetics will change and the removal percent will decrease.

The pseudo-first-order [29] and pseudo-second-order [30] kinetic models were employed to gather information on the adsorption dynamics [31]; the equations presented in Table 1. The applicability of the models via linear regression can be estimated by presenting the experimental data in plots: a linear plot of log(*q_e_* − *q_t_*) against t indicates better fit with pseudo-first-order model while a linear plot of *t*/*q_t_* versus t presents better fit with pseudo-second-order model (Figure S1) [7]. The experimental *q_t_* and the corresponding theoretical values, which were obtained by inserting the linearized parameters from Table 1 in the kinetic models are displayed in Figure S2. The kinetic constants and the linear *R*^2^ values from the models are compiled in Table 1. The linear *R*^2^ values corresponding to pseudo-first-order model all fall under 0.8 whereas the *R*^2^ values corresponding to pseudo-second-order model are all above 0.99 (Table 1 and Figure S1). This suggests that the pseudo-second-order is more applicable than pseudo-first-order model in presenting the kinetics of phosphate adsorption onto Zn(II)–CTS.

**Figure 7:**
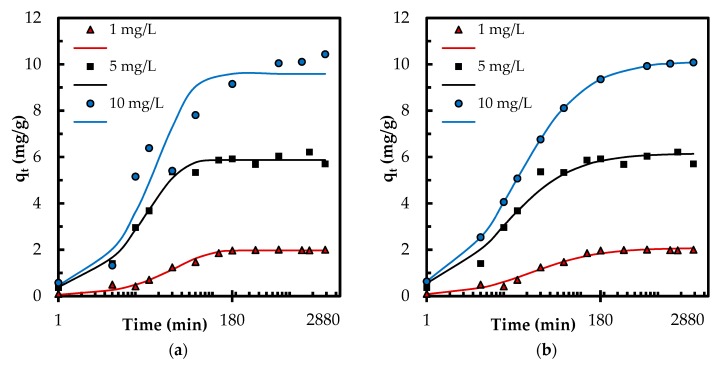
(**a**) Theoretical *q_t_* (lines) by pseudo-first-order kinetic model; and (**b**) theoretical *q_t_* (lines) by pseudo-second-order kinetic model determined by nonlinear regression compared to experimental *q_t_* (markers).

In addition, the normalized standard deviation Δ*q* is determined for quantitative comparison of the model applicability, given in Table 1.

(3) $$\Delta q=\sqrt{\frac{\sum {\left[\left(q_{exp}-q_{cal}\right)/q_{exp}\right]}^{2}}{n-1}}$$

where *n* is the number of data points. Even though the linear regression provided high *R*^2^ values, the corresponding Δ*q* values (Table 1) and Figure S2 indicate a poor performance of linear regression for determination of best fitting kinetic models. Therefore, a nonlinear regression has been employed to further explore the kinetic data and confirm the best fitting kinetic model (Table 1 and Figure 7). The coefficient of determination *R*^2^ is employed to find out the best-fitting model via nonlinear regression.

(4) $$R^{2}=\frac{\sum_{i=1}^{p}{\left(q_{exp}-\bar{q_{calc}}\right)}^{2}}{\sum_{i=1}^{p}{\left(q_{exp}-\bar{q_{calc}}\right)}^{2}+\sum_{i=1}^{p}{\left(q_{exp}+\bar{q_{calc}}\right)}^{2}}$$

where $\bar{q_{calc}}$ is the average of *q_calc_*. The experimental data (*q_exp_*) aligned better with the theoretical data (*q_cal_*) obtained by nonlinear regression of pseudo-second-order model (Figure 7) that is consistent with corresponding nonlinear *R*^2^ and Δ*q* values. This indicates that the adsorption may be involved in chemisorption [6] and is influenced by the characteristics of both the adsorbent and adsorbate [28]. The phosphate adsorption also followed pseudo-second-order kinetics in other studies [3,4,5,32].

**Figure 8:**
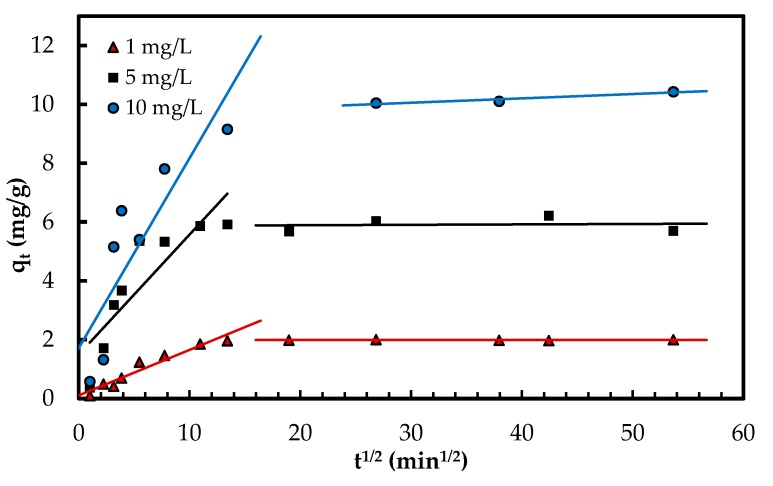
Phosphate adsorption steps onto Zn(II)–CTS bio-sorbent determined with the intra-particle diffusion model.

Intra-particle diffusion mechanism was explored according to Weber and Morris model [33] using the equation given in Table 1. In this model, the C constant represents the thickness of the boundary layer [7]. The results are compiled in Table 1. The multi-linear plot of *q_t_* versus *t*^1/2^ (Figure 8) suggests several phases occur in the adsorption process and the pore diffusion is not the only rate-limiting stage [7,33]. During the first phase, the phosphate oxyanions are transferred onto the surface of Zn(II)–CTS complexes from the solution (boundary layer phase). During the second and slower phase, the anions are transported to the pores of Zn(II)–CTS particles where intra-particle diffusion is the rate-limiting step. As discussed in earlier sections, the surface charge of the bio-sorbent is mainly of positive or neutral charge in the studied pH range, therefore the transfer of phosphate anions from the solution to the surface of the bio-sorbent can take place faster and more easily. In some cases, there is a third and final stage which can be considered as the equilibrium phase in which intra-particle diffusion starts slowing down because of the very dilute concentration of adsorbate remained in the liquid phase [34,35]. In Figure 8, the first two phases of adsorption are visible. 

#### 3.2.4. Adsorption Isotherm 

**Figure 9:**
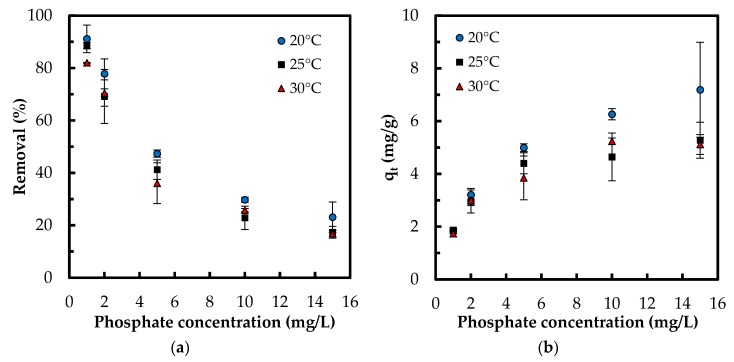
Effect of temperature on: (**a**) phosphate removal percentage; and (**b**) adsorption capacity (mg/g) (Experimental conditions: 0.5 g/L Zn(II)–CTS dose; pH 4; 24 h contact time).

The isotherm tests were conducted in five phosphate concentrations of 1, 2, 5, 10 and 15 mg/L at 20, 25, and 30 °C. When comparing the results of varying temperature, both the removal percentage and the adsorption capacity improved with a decrease in temperature (Figure 9). For instance, at concentration 5 mg/L, adsorption capacity increased from 3.9 to 5 mg/g and the removal percentage increased from 36% to 47.4% with decreasing temperature from 30 to 20 °C, respectively. This indicates that lower temperatures favor the adsorption process. When comparing the results at a constant temperature, while the adsorption capacity increased with increasing PO_4_^3−^ concentration, the removal percentage diminished. For instance, at 20 °C, adsorption capacity increased from 1.8 to 7.2 mg/g while the removal percentage decreased from 91% to 23% with increasing phosphate concentration from 1 to 15 g/L, respectively. Lower removal percentage at higher phosphate concentrations can be caused from the saturation of adsorptive sites. The equilibrium data were examined with Langmuir, Freundlich, and Sips isotherms. While the Langmuir model hypothesizes a homogenous monolayer adsorption without molecule interaction, Freundlich isotherm assumes multilayer adsorption with molecule interaction [36]. The Sips isotherm, combining the basics of Langmuir and Freundlich isotherms, represents systems where an adsorbed molecule can be involved with more than one adsorptive site [15]. At lower adsorbate concentrations, this model turns to a Freundlich model, while, at higher concentrations, it gives the monolayer coverage characteristic of Langmuir isotherm. Giles et al. [37] have presented four main types of capacity curves, C, L, H and S. Herein, the adsorption capacity curve resembled the “L” isotherm without a strict plateau, i.e., it does not reach a point of limited adsorption capacity. For the “L” capacity curve, the Freundlich isotherm was found to be the most suitable [37]. 

The calculated parameters via linear regression along with the linearized isotherm equations are compiled in Table 2. The unitless Langmuir separation factor *R_L_* shows the favorability of the adsorption; the values between 1 and 0 suggest favorable adsorption [7]. The linear *R*^2^ values for Langmuir model were slightly higher than those of Freundlich model. However, the calculated values for *q_e_* with linearized Freundlich isotherm showed a better fit with experimental *q_e_* (Δ*q* values in Table 2 and Figure S3).

(5) $$R_{L}=\frac{1}{1+K_{L}C_{0}}$$

**Figure 10:**
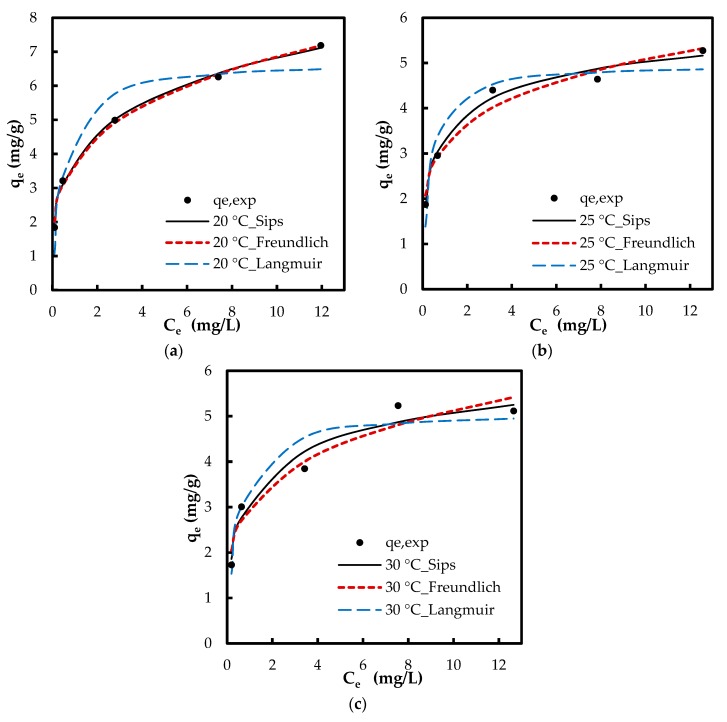
Experimental *q_e_* (markers) and theoretical *q_e_* (lines) via nonlinear regression of isotherm models at: (**a**) 20 °C; (**b**) 25 °C; and (**c**) 30 °C.

Even though linear regression is frequently employed to determine the best fitting isotherm, the error structure can alter upon linearizing the nonlinear equations. Based on the way the isotherm is linearized, the error distribution may change for either the worse or the better. Nonlinear regression is more suitable for the determination of the isotherm parameters, which can prevent such errors. Furthermore, the linear regression is inapplicable for isotherms with more than two adjustable parameters, e.g., Sips [15]. Therefore, the isotherm parameters were also determined by nonlinear regression. For nonlinear method, a trial and error approach was employed by minimizing the error between experimental data and calculated values. The calculated isotherm parameters via nonlinear regression are compiled in Table 3. The nonlinear *R*^2^ values for Freundlich isotherm via nonlinear regression were higher when compared with those of the linear *R*^2^ values. This indicates the error distribution altered to the worse while fitting the experimental data in linearized Freundlich model. The Freundlich *R*^2^ values were higher when compared with those of Langmuir *R*^2^ values (nonlinear regression). Figure 10 depicts the experimental *q_e_* and the predicted *q_calc_* by the isotherm models via nonlinear modeling. The Δ*q* values based on Freundlich model were also lower compared with those of Langmuir isotherm via both linear and nonlinear regressions. Figure 10 and Table 3 clearly suggest that the adsorption isotherm of phosphate onto Zn(II)–CTS is better fit to a Freundlich model than a Langmuir model, yet this is the Sips model indicating the best fit. The fitting of the experimental data to the Sips model indicates that phosphate adsorption takes place on homogeneous–heterogeneous surface of the bio-sorbent. 

#### 3.2.5. Adsorption Thermodynamics

The adsorption thermodynamics, the change of Gibb’s free energy (∆*G*), the change of entropy (∆*S*), and the change in enthalpy (∆*H*) [38] are defined by:

(6) $$\Delta G=-RT\;\ln K_{0}$$

(7) ΔG=ΔH−TΔS

where *R* is the universal gas constant (8.314 J/K·mol), *T* (K) the temperature, and *K*_0_ the unitless thermodynamic equilibrium constant [39]. The *K*_0_ is extrapolated from the plot of ln(*q_e_*/*C_e_*) versus *C_e_* (Figure S4) [39]. The thermodynamic parameters are tabulated in Table 4.

The negative values of ∆*G* indicate the spontaneous adsorption of phosphate onto Zn(II)–CTS. Table 4 shows the negative value of ∆*G* increases with decreasing temperature, which is consistent with the isotherm results showing a more favorable adsorption at lower temperatures. The negative enthalpy change confirms the exothermic characteristic of adsorption. The heat evolved in the adsorption reveals the physical and chemical nature of the reaction. The value of ∆*H* (−37.86 kJ/mol) suggests the phosphate adsorption onto Zn(II)–CTS is a physicochemical process rather than a purely physical or chemical adsorption [26,40]. The negative ∆*S* indicates the randomness decreases at the adsorption interface. Similar results have been reported in previous studies [41,42]. Worch [38] argues that a negative change in entropy is caused by the immobilization of adsorbate in the system. It can also indicate the fast adsorption of phosphate on the adsorptive sites [41]. 

#### 3.2.6. Effect of Coexisting Ions

**Figure 11:**
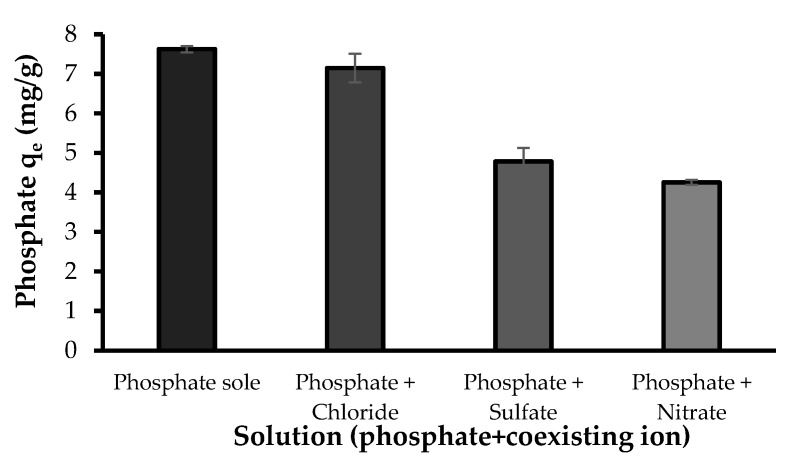
The effect of coexisting ions on phosphate adsorption (Experimental conditions: 5 mg/L phosphate concentration; 5 mg/L coexisting ion concentration; 0.5 g/L Zn(II)–CTS dose; pH 4; 24 contact time).

Natural waters usually contain other anions competing for the adsorptive sites with phosphate. The competing effect of coexisting anions on phosphate adsorption by the developed bio-sorbent was studied with NO_3_^−^, SO_4_^2−^ and Cl^−^ ions. Of these ions, chloride showed a minor effect, while nitrate and sulfate showed competing effect on the adsorption of phosphate (Figure 11). The equilibrium capacity of the bio-sorbent for phosphate in the absence of other ions was 7.63 mg/g while the phosphate adsorption in the presence of NO_3_^−^, SO_4_^2−^ and Cl^−^ were 4.26, 4.79 and 7.15 mg/g, respectively. These results agree with previously reported studies on phosphate adsorption [3,4]. The sensitivity of the phosphate adsorption to the presence of background ions, e.g., SO_4_^2−^ and NO_3_^−^, can indicate the outer-sphere complexation of phosphate with the bio-sorbent. Liu and Zhang [3] reported a notable reduction in phosphate uptake by modified chitosan beads in the presence of SO_4_^2−^, while Cl^−^ and NO_3_^−^ slightly reduced phosphate adsorption. It was explained that SO_4_^2−^ ions could more likely link with the functional groups on the surface of the composite, which in turn reduced the available active sites and hindered phosphate uptake. In addition, the accumulation of sulfate ions on the surface of the composite could contribute in forming a negatively charged surface, leading to an increasing repulsive force against phosphate oxyanions and hence a decreasing phosphate uptake. In the case of competing effect of nitrate, Seliem et al. [6] reported that the presence of NO_3_^−^ significantly reduced the phosphate adsorption onto the composite of MCM-41 silica with rice husk and they attributed this effect to the initial pH of the solutions (pH 9.56). Their study showed that by maintaining a constant pH of 6, no obvious shift was noticed in the removal of phosphate, especially in the presence of nitrate molecules. Herein the nitrate molecules might hinder the adsorption of phosphate through mechanisms including the accumulation of nitrate anions on the surface of Zn(II)–CTS and formation of a negatively charged surface. Based on the results observed in this study and those previously reported, it can be inferred that: (I) The binding affinity of these ions for the adsorptive sites of the developed bio-sorbent are comparable with those of phosphate. (II) These ions can compete with phosphate for the adsorptive sites. (III) The mechanisms involved in the adsorption of phosphate and the other two ions onto the developed bio-sorbent are similar [43].

### 3.3. Mechanisms of Zn(II)–CTS Complexation and Phosphate Adsorption

CTS provided limited affinity for the oxyanions of phosphate as showed in Figure 4, which can be due to the p*K*_a_ values of its electron-donating groups. Metal cations, e.g., Zn(II), however, are able to bind to CTS at different electron-donating sites, e.g., amine –NH_2_, on its chain. The complexed metal ion can then coordinate other ligands, providing adsorptive sites for phosphate on the CTS matrix that previously were unavailable, which is confirmed with the results presented in Figure 4. The mechanisms of metal ions complexation with CTS can be categorized in two categories of: (I) monodentate pattern; and (II) bidendate pattern [12,20]. In the former mechanism (I), the metal ions bind to one functional groups on CTS chain, while in the latter type (II) the metal ions bind to two or more functional groups, e.g., amino and hydroxyl groups, on one or more CTS chains so that act as a bridge between the chains [20]. Wang et al. [20] developed Zn(II)–CTS complexes for antibacterial application and noted that the complexes prepared with different zinc/CTS ratios were found to coordinate different amount of the metal ion and indicated diverse characteristics. The importance of pH and metal ion/CTS ratio in coordinating the metal ions on CTS structure was also reported for the Cu(II)–CTS complexes. Large Cu(II) loading changes the complexation mechanism from solely type (I) to a combination of types (I) and (II). It was also reported that the pH range ˂~5.5 mainly led to the formation of type (I) and exceeding pH values created more of type (II) [44]. The formation of metal ion–CTS complexation can be explained through Lewis acid–base theory, where the metal ion (M^2+^) plays the role of the acid by accepting the electron pairs provided by CTS as the base. The reaction equations are written as follows:

CTS−NH_2_ + H_3_O^+^ → CTS−NH_3_^+^ + H_2_O (8)

**Figure 12:**
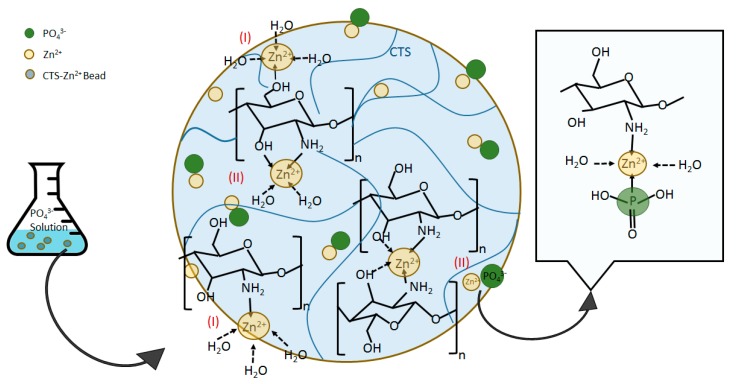
Possible structures of Zn(II)–CTS complexation and corresponding mechanism for phosphate uptake.

CTS−NH_3_^+^ + M^n+^ + H_2_O → (CTS−NH_2_−M)^n+^ + H^+^ + H_2_O (9)

where (CTS−NH_2_−M)^n+^ represents the metal ion–CTS complexes. Herein, M^n+^ is Zn^2+^ (Zn(II)). The FT-IR analysis showed that the –NH_2_ and –OH functional groups of CTS are involved in the complexation of CTS with Zn(II) in the bio-sorbent. The potential molecular structures of Zn(II)–CTS complexation are depicted in Figure 12. 

The type (I) complexation has been reported as the main mechanism favoring the uptake of phosphate Cu(II)–CTS complexes [12]. Some extent of phosphate uptake might also occur through the type (II) mechanism, yet, the magnitude of this uptake is minor in comparison with those of type (I) [12]. One explanation for this preference may lie in electrostatic interactions: electrons are more likely in a “diffused state” in complexation type (II) as they are distributed between different monomers and chains of CTS, whereas, in complexation type (I), electrons are configured in a more “concentrated state” so that electrostatic forces are strong enough to attract phosphate anions [12,45]. Besides, the involvement of two CTS chains in complexation type (II) may obstruct phosphate from adsorbing. Accordingly, the mechanism of phosphate uptake by the Zn(II)–CTS bio-sorbent proposed here is illustrated in Figure 12.

### 3.4. Evaluation of Zn(II)–CTS Bio-Sorbent

The adsorption capacities achieved by Zn(II)–CTS are compared with those of earlier reports on engineered adsorbents including zirconium–modified chitosan (ZCB) [3], zinc ferrite [46], magnetic iron oxide nanoparticles [47], ZnCl_2_–activated carbon [48], titanium dioxide [49], synthetic iron oxide coated sand [50] and magnetic illite clay [51], which are compiled in Table 5. The adsorption capacity of Zn(II)–CTS was superior or comparable to the other adsorbents. For instance, magnetic iron, ZnCl_2_–activated carbon, zinc ferrite, and synthetic iron oxide coated sand provided lower maximum adsorption capacities compared with those of observed in the present study. When comparing different adsorbents studied for phosphate removal, the differences in operational conditions need to be taken into account. Unlike the studies conducted with higher phosphate concentrations, e.g., ZCB (Table 5), Zn(II)–CTS was studied with relatively lower, yet, more environmentally relevant phosphate concentrations, which most likely explains the difference in maximum capacities. 

## 4. Conclusions

This study developed zinc(II)–chitosan bio-sorbents for phosphate removal from aqueous solution. The effect of operational parameters, e.g., pH, dose and contact time, on the adsorption phosphate by the complexes was explored in detail. The optimum dose of the bio-sorbent was found to be 0.5 mg/L. It was observed that lower pH values favor the adsorption of phosphate. A rapid adsorption was observed within the first 3 h of contact time for all three studied concentrations. The temperature study revealed that the adsorption process was more successful in lower temperatures and it was exothermic and spontaneous in nature. The study of co-existing ions revealed that Cl^−^ shows minor effect on phosphate removal, whereas NO_3_^−^ and SO_4_^2−^ show competing effect. Pseudo-second-order model was more applicable to kinetics study. The intra-particle diffusion study revealed that at least two steps were involved in the adsorption process, the boundary layer stage and the intra-particle diffusion stage. The adsorption isotherm was well fitted with Freundlich and Sips isotherms. CTS is a unique adsorbent, a completely natural and biodegradable polymer. Along with studies on the antimicrobial activity, thermal degradation, and pyrolysis characteristics of CTS complexes with zinc(II) [20,21], this study provides deeper understanding of the zinc(II)–CTS as a bio-sorbent for more accurate application towards specific target, e.g., phosphate removal. 

## Figures and Tables

**Table 1:** Calculated parameters for kinetic models via linear and nonlinear regression.

*C*_0_ (mg/L)	*q_e(exp)_*	Pseudo-first-order				Pseudo-second-order				Intra-particle diffusion	
		Linear regression									
		$\ln\left(q_{e}-q_{t}\right)=\ln\;q_{e}-k_{1}t$				$\frac{t}{q_{t}}=\frac{1}{k_{2}q_{e}^{2}}+\frac{t}{q_{e}}$				$q_{t}=k_{p}t^{\frac{1}{2}}+C$	
		*q_e(cal)_*	*k*_1_	*R*^2^	Δ*q*	*q_e(cal)_*	*k*_2_	*R*^2^	Δ*q*	*k_p_*	*C*
1	1.99	0.61	0.003	0.66	3.85	2.01	0.029	1.00	0.90	0.030	0.875
5	6.34	1.85	0.004	0.55	3.27	5.85	1.476	0.998	1.03	0.067	3.541
10	10.44	4.64	0.002	0.76	3.22	10.46	0.004	1.00	0.48	0.150	4.312
		**Nonlinear regression**									
1	1.99	1.97	0.029	0.98	0.34	2.08	0.020	0.981	0.17	-	-
5	6.34	5.87	0.067	0.99	0.06	6.16	0.016	0.992	0.15	-	-
10	10.44	9.59	0.048	0.91	0.25	10.13	0.007	0.984	0.20	-	-

Note: *k*_1_ pseudo-first-order rate constant (min^−1^); *t* time (min); *q_e_* and *q_t_* phosphate adsorbed per adsorbent mass at equilibrium and at time *t* (mg/g); *k*_2_ pseudo-second-order rate constant (g/mg·min); *k_p_* intraparticle diffusion constant (mg/g·min^1/2^); *C* boundary layer thickness constant (mg/g).

**Table 2:** Calculated isotherm parameters via linear regression.

*T* (K)	Langmuir					Freundlich			
	$\frac{C_{e}}{q_{e}}=\frac{1}{q_{max}}C_{e}+\frac{1}{K_{L}q_{max}}$					$\log q_{e}=\log K_{F}+\frac{1}{n}\log C_{e}$			
	*q_max_*	*K_L_*	*R_L_*	*R*^2^	Δ*q*	*K_F_*	1/*n*	*R*^2^	**Δ*q***
293	7.37	1.32	0.05–0.43	0.99	0.31	3.72	0.27	0.99	0.04
298	5.33	1.85	0.03–0.34	0.99	0.24	3.12	0.22	0.98	0.06
303	5.40	1.56	0.04–0.38	0.99	0.18	2.92	0.25	0.95	0.1

Note: *C_e_* equilibrium phosphate concentration; *K_L_* Langmuir thermodynamic constant (L/mg); *R_L_* Langmuir separation factor; *q_e_* adsorption capacity in equilibrium; *q_max_* calculated maximum adsorption capacity at each temperature; *K_F_* Freundlich thermodynamic constant ((mg/g)·(L/mg)^1/*n*^); 1/*n* Freundlich’s intensity factor.

**Table 3:** Calculated isotherm parameters via nonlinear regression.

*T* (K)	Langmuir				Freundlich				Sips				
	$q_{e}=\frac{q_{max}K_{L}C_{e}}{1\;+\;K_{L}C_{e}}$				$q_{e}=K_{F}C_{e}^{\frac{1}{n}}$				$q_{e}=\frac{q_{s}K_{s}C_{e}^{ns}}{1\;+\;K_{S}C_{e}^{ns}}$				
	*q_max_*	*K_L_*	*R*^2^	Δ*q*	*K_F_*	1/*n*	*R*^2^	Δ*q*	*q_s_*	*K_s_*	*n_S_*	*R*^2^	Δ*q*
293	6.75	2.11	0.92	0.23	3.77	0.26	1.00	0.05	17.1	0.29	0.34	1.00	0.03
298	4.98	3.19	0.93	0.16	3.17	0.20	0.97	0.07	6.94	0.94	0.33	0.99	0.04
303	5.12	2.25	0.93	0.11	3.01	0.23	0.95	0.12	7.26	0.77	0.48	0.96	0.08

Note: *q_s_* Sips maximum capacity (mg/g); *K_S_* Sips equilibrium constant ((L/mg)*^ns^*); *n_S_* Sips exponent.

**Table 4:** Calculated thermodynamic parameters.

*T* (K)	∆*G* (kJ/mol)	∆*H* (kJ/mol)	∆*S* (J/K∙mol)
293	−5.27	−37.86	−111.32
298	−4.60		
303	−4.15		

**Table 5:** Phosphate adsorption on different engineered adsorbents.

Adsorbent	*C*_0_ (mg/L)	*q_max_* (mg/g)	pH	Reference
Zn(II)–CTS	5	7.37	4	Present
Zn(II)–CTS	5	6.3	natural	Present
Chitosan	5	1.45	natural	Present
Chitosan	5	4.75	4	Present
Naturally iron oxide coated sand	5–30	0.88	5	[50]
Synthetic iron oxide coated sand	5–30	1.50	5	[50]
Zinc ferrite	5	5.23	-	[46]
Magnetic illite clay	10–100	5.48	-	[51]
ZCB	5–50	60.60	4	[3]
Quaternized chitosan beads	1000	59.00	-	[5]
TiO_2_	-	2.63	5	[49]
ZnCl_2_–carbon	10	5.10	-	[48]
Magnetic iron	2–20	5.03	-	[47]

## Supplementary Materials

The following are available online at www.mdpi.com/2073-4360/10/1/25/s1, Figure S1: The linear plot of *t*/*q_t_* versus t for the determination of psodo-second-order kinetic model parameters via linear regression. Figure S2: (a) Theoretical *q_t_* (lines) by pseudo-first-order kinetic model; and (b) theoretical *q_t_* (lines) by pseudo-second-order kinetic model compared to experimental *q_t_* (markers). Figure S3: (a) Theoretical values of *q_e_* (lines) from Langmuir isotherm model; and (b) theoretical values of *q_e_* (lines) from Freundlich isotherm model compared to experimental *q_e_* (markers). Figure S4: The plot of ln(*q_e_*/*C_e_*) versus *C_e_* for determination of *K*_0_.
