# Supplementary material for: Chitosan–Zinc(II) Complexes as a Bio-Sorbent for the Adsorptive Abatement of Phosphate: Mechanism of Complexation and Assessment of Adsorption Performance
Source: Polymers (Basel). 2017 Dec 25;10(1):25. doi: 10.3390/polym10010025 (PMC6414967; doi:10.3390/polym10010025)
Supplement: Supplementary file 1 [file polymers-10-00025-s001.pdf]

Supplementary Information:

Chitosan–Zinc(II) Complexes as a Bio-Sorbent for the Adsorptive Abatement of Phosphate: Mechanism of Complexation and Assessment of Adsorption Performance

Maryam Roza Yazdani [1,*], Elina Virolainen [1], Kevin Conley [2] and Riku Vahala [1]

[1] Water and Wastewater Engineering Research Group, School of Engineering, Aalto University, P.O. Box 15200, FI-00076 Aalto, Finland; elina.virolainen@gmail.com (E.V.); riku.vahala@aalto.fi (R.V.)

[2] COMP Centre of Excellence, Department of Applied Physics, School of Science, Aalto University, FI-00076 Aalto, Finland; kevin.conley@aalto.fi

* Correspondence: roza.yazdani@aalto.fi; Tel.: +358-4074-2014

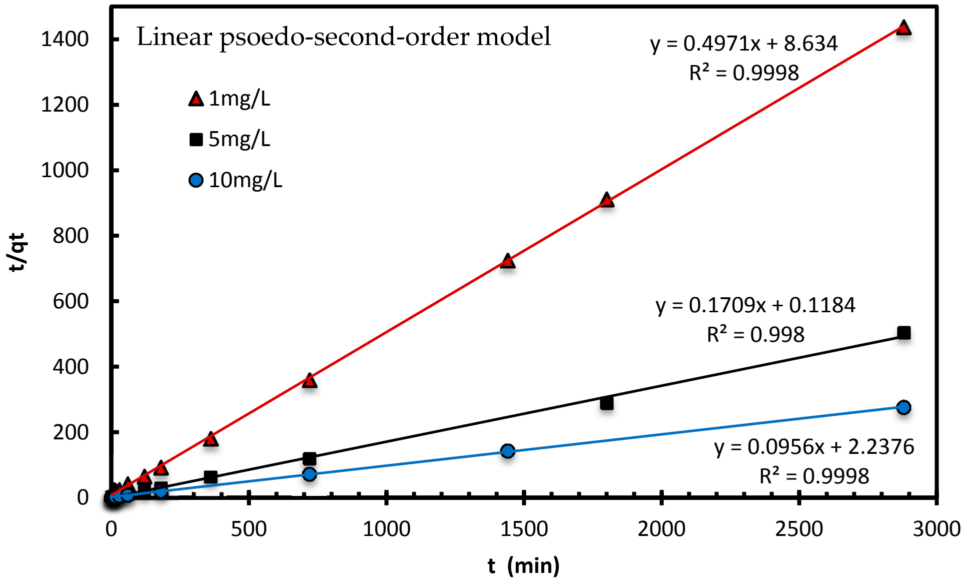

Figure S1. The linear plot of t/q_t versus t for the determination of psodo-second-order kinetic model parameters via linear regression.

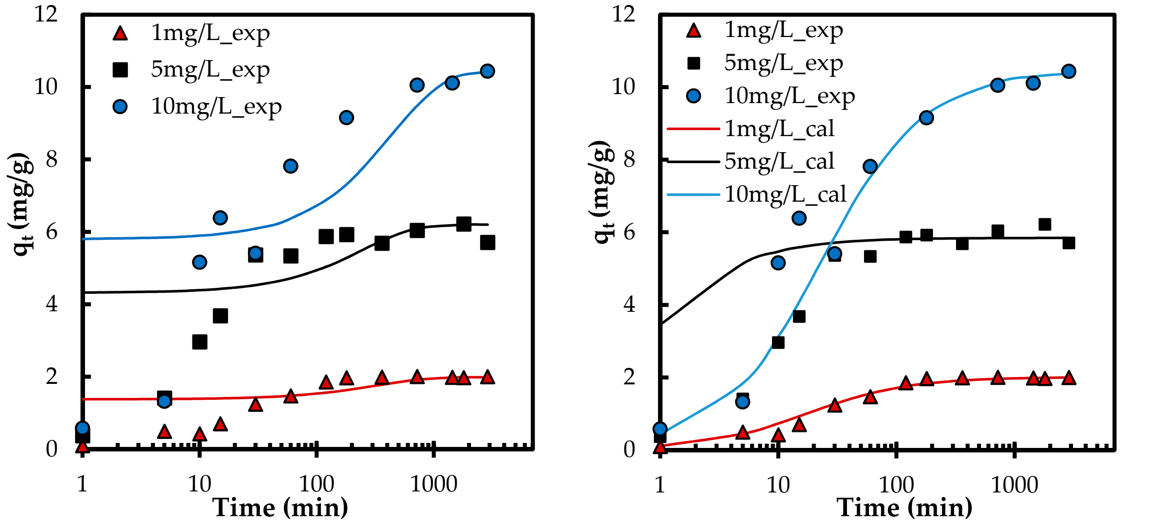

(a) (b)

Figure S2. (**a**) Theoretical q_t (lines) by pseudo–first–order kinetic model and (**b**) theoretical q_t (lines) by pseudo–second–order kinetic model compared to experimental q_t (markers).

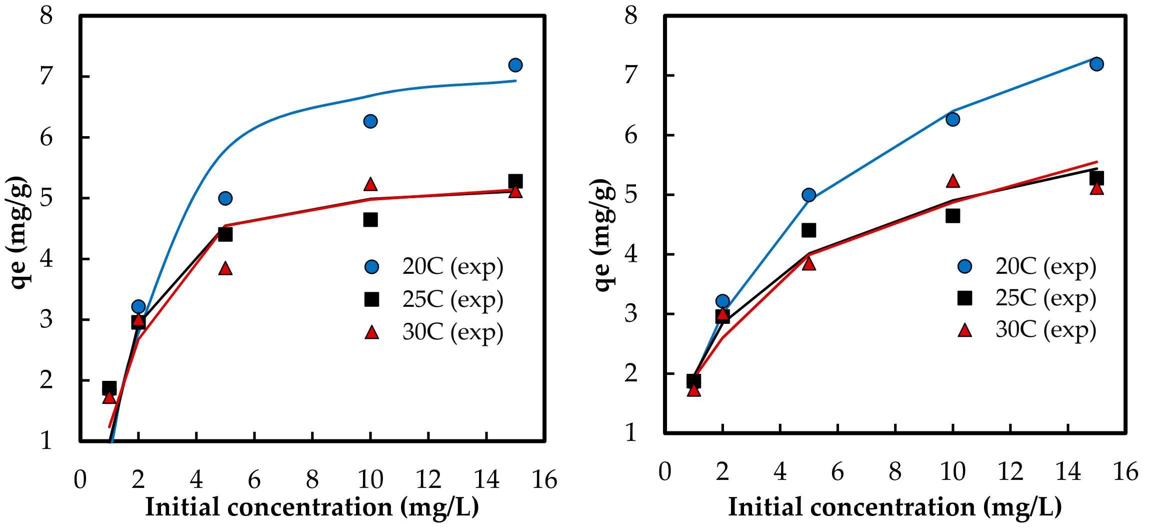

(a) (b)

Figure S3. (**a**) Theoretical values of q_e (lines) from Langmuir isotherm model and (**b**) theoretical values of q_e (lines) from Freundlich isotherm model compared to experimental q_e (markers).

Non-linear approach for isotherm studies:

The non-linear isotherm parameters of the Sips model were obtained by minimizing the error values through a "trial and error" approach via the Solver "add-in" in Microsoft Excel-2016 [1–4].

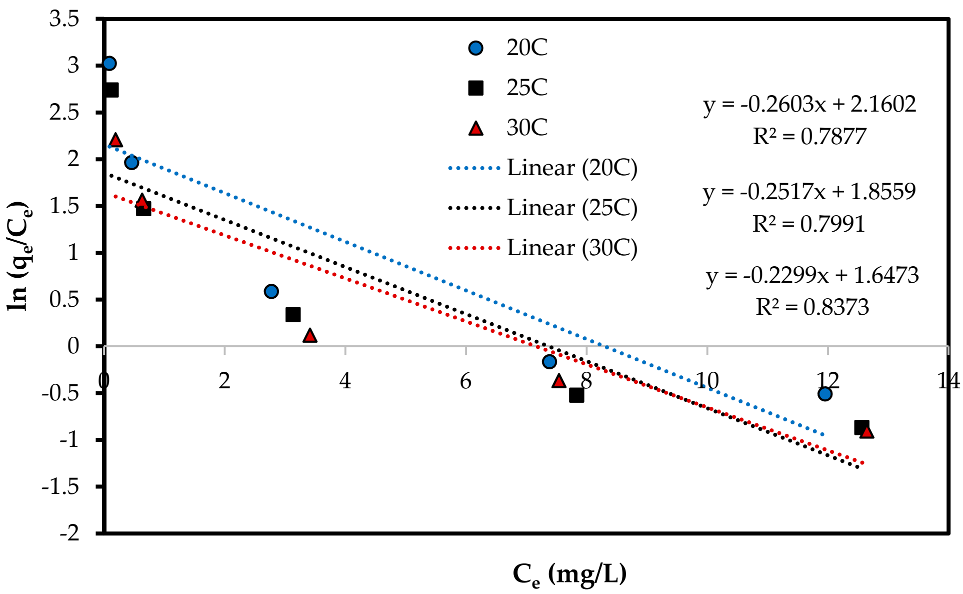

Figure S4. The plot of $ln(q_e/C_e)$ versus C_e for determination of K_0.

Reference

1. Brdar, M.; Sciban, M.; Takaci, A.; Dosenovic, T. Comparison of two and three parameters adsorption isotherm for Cr(VI) onto kraft lignin. *Chem. Eng. J.* **2012**, *183*, 108–111.
2. Dubey, S.; Gusain, D.; Sharma, Y.C. Kinetic and isotherm parameter determination for the removal of chromium from aqueous solutions by nanoalumina, a nanoadsorbent. *J. Mol. Liq.* **2016**, *219*, 1–8.
3. Kumar, K.V. Comparative analysis of linear and non-linear method of estimating the sorption isotherm parameters for malachite green onto activated carbon. *J. Hazard. Mater.* **2006**, *B136*, 197–202.
4. Sips, R. On the structure of a catalyst surface. *J. Chem. Phys.* **1948**, *16*, 490–495.
